# *Notes from the Field:* Elevated Atmospheric Lead Levels During the Los Angeles Urban Fires — California, January 2025

**DOI:** 10.15585/mmwr.mm7405a4

**Published:** 2025-02-20

**Authors:** Haroula D. Baliaka, Ryan X. Ward, Roya Bahreini, Ann M. Dillner, Armistead G. Russell, John H. Seinfeld, Richard C. Flagan, Paul O. Wennberg, Nga L. Ng

**Affiliations:** ^1^California Institute of Technology, Pasadena, California; ^2^University of Riverside, Riverside California; ^3^University of California Davis, Davis, California; ^4^Georgia Institute of Technology, Atlanta, Georgia.

**Summary**

**What is already known about this topic?**

Smoke is a complex mixture of gases and airborne particulate matter; urban fires and conventional wildfires emit different air pollutants. The Atmospheric Science and Chemistry mEasurement NeTwork (ASCENT), a new, advanced air quality measurement network, provides real-time measurements of the chemical constituents in fine particulate matter (PM_2.5_).

**What is added by this report?**

During the January 2025 Los Angeles fires, ASCENT recorded an approximate 110-fold increase in PM_2.5_ lead levels compared with values from the previous few days.

**What are the implications for public health practice?**

Urban fires emit air pollutants that pose risks different from those of conventional wildfires. It is important for epidemiologic studies to consider PM_2.5 _composition when assessing the impacts of urban fire smoke exposure. Health officials should communicate protective measures to the public (monitor air quality forecasts and follow guidance by local emergency management officials).

On January 7, 2025, the Eaton Canyon and Palisades fires blazed across the Los Angeles region, driven by exceptionally dry conditions and Santa Ana wind gusts approaching 100 mph (161 kph). The fires spread rapidly into densely populated neighborhoods along the wildland-urban interface, destroying approximately 16,000 structures. As of February 10, 2025, a total of 29 deaths had been identified.* In addition to the deaths and destruction of property, wildfires emit a complex mixture of air pollutants and contribute to elevated concentrations of fine particulate matter (PM_2.5_; particulate matter with a diameter <2.5 *μ*m), degrading air quality many miles downwind. Exposure to wildfire PM_2.5_ has been linked to adverse health effects including increased asthma cases, respiratory symptoms, aggravated respiratory diseases, and increased overall mortality (*1*–*3*). Unlike conventional wildfires that primarily burn natural fuels (e.g., grasslands or forests), the Eaton Canyon and Palisades fires ignited significant portions of the built environment, in which painted surfaces, pipes, vehicles, plastics, electronic equipment, and the structures themselves became the fuel. This widespread combustion of synthetic materials has increased concerns about the toxicity of PM_2.5_, because a large proportion of the structures affected by the fires were built before 1978, when use of leaded paint was still common. This report focused on measuring airborne PM_2.5_ lead during the Los Angeles urban fires.

## Investigation and Outcomes

The Atmospheric Science and Chemistry mEasurement NeTwork (ASCENT)^†^ is a new, nationwide, multi-institutional initiative funded by the National Science Foundation, to provide continuous measurements of PM_2.5_ chemical components (organics, inorganics, metals, and black carbon) across 12 sites in the United States, including seven urban and five remote or rural areas.^§^ All ASCENT sites were operating and sampling ambient air as of May 2024.

The Los Angeles ASCENT site in Pico Rivera, approximately 14 miles (23 kilometers) south of the Eaton Canyon fire, has been operating since July 2023. During and immediately after the Los Angeles fires, southward winds transported the fire plume to the ASCENT site. Hourly PM_2.5_ lead measurements recorded during and after the fires were reviewed to assess their contribution to atmospheric lead levels. Because this analysis consists of a review of routinely collected environmental data and does not include human subjects, human subjects review was not required by the authors’ institutions.

During January 2–6, 2025, the average PM_2.5_ lead concentration recorded at the Los Angeles ASCENT site was 0.00068 *μ*g/m^3^. From January 8 to January 11, PM_2.5_ lead concentration increased approximately 110 times with an average concentration of 0.077 *μ*g/m^3^ (Figure). Recorded PM_2.5_ lead concentration peaked at approximately 0.5 *μ*g/m^3^ on January 9. By the evening of January 11, PM_2.5_ lead concentration had returned to levels similar to those before the fire. The presence of heavy metals such as lead is not unusual in urban fire emissions, particularly in California, where legacy pollutants from older infrastructure, industrial sources, and soils can be remobilized during fires (*2*,*4*). For example, during the 2018 Camp fire, monitors recorded ambient PM_2.5 _lead concentrations that averaged 0.13 *μ*g/m^3^ during a period of 17 hours (*2*).

**FIGURE:**
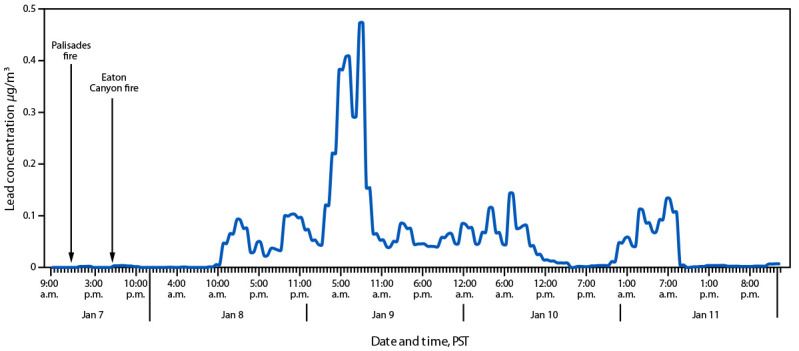
Hourly lead concentrations*^,^^†^ of particulate matter <2.5 *μ*m in diameter at the Los Angeles Atmospheric Science and Chemistry mEasurement NeTwork site relative to the start of the Palisades and Eaton Canyon fires — Pico Rivera, California, January 7–12, 2025 * *μ*g/m^3^. ^†^ The National Ambient Air Quality Standard for lead in total suspended particles over a 3-month rolling average is 0.15 *μ*g/m^3^.

Few data illustrate the health effects of lead from inhalation compared with other exposure routes. The ASCENT real-time measurements of airborne lead and other chemical constituents in PM_2.5_ provide valuable PM_2.5_ chemical composition data that can be combined with health data to examine health effects of individual smoke components from the Los Angeles fires.

## Preliminary Conclusions and Actions

Lead is a toxic air contaminant that is distributed in multiple human tissues and accumulates in teeth and bones; it affects nearly every organ system, posing significant health risks, particularly for children, who are more vulnerable to its neurodevelopmental effects (*2*,*3*,*5*). Regulatory efforts, especially the U.S. Clean Air Act of 1970, have resulted in a sharp decline in airborne lead levels during the past 45 years.^¶^ The current National Ambient Air Quality Standard for lead in total suspended particles over a 3-month rolling average is 0.15 *μ*g/m^3^.** Measures including removing lead from gasoline and leaded pipes and the banning or limiting of lead in consumer products, such as residential paint, have led to a 97% decrease in airborne lead concentrations in the United States since 1980 (*5*). However, unlike chronic lead exposure, which has been widely studied, the health effects of brief, elevated lead exposures, such as those described in this report, are not well understood. Additional health research is needed, because airborne lead levels alone do not necessarily indicate exposure.

PM_2.5_ is not a single entity but comprises a complex mixture of chemical components with dynamic size distributions, temporal and spatial variations, and toxicity. Whereas the health effects of PM_2.5_ exposure are well documented, studies assessing which sources, chemical compounds, and sizes of particles contribute to health effects are lacking. ASCENT fills in this gap by providing high time-resolution and chemical composition measurements of PM_2.5_ across dynamic size ranges with advanced air quality measurement technologies. The new availability of real-time measurements of the many chemical constituents in PM_2.5_, and time-resolved particle size distributions in diverse U.S. locations, has the capacity to improve understanding of health effects associated with particulate matter exposure and contribute to building a foundation for protecting public health.

## Footnotes

* https://www.fire.ca.gov/incidents/ (Accessed February 10, 2025).

†
https://ascent.research.gatech.edu/

§ The seven urban areas are Atlanta, Georgia; Denver, Colorado; Houston, Texas; Los Angeles, California; New York, New York; Pittsburgh, Pennsylvania, and Riverside, California. The five remote or rural areas include Alaska, Cheeka Peak/Makah in Washington, and the Great Smoky Mountains, Joshua Tree, and Yellowstone National Parks.

¶
https://www.epa.gov/laws-regulations/summary-clean-air-act

**
https://www.epa.gov/lead-air-pollution/national-ambient-air-quality-standards-naaqs-lead-pb

## References

[1] Aguilera R, Corringham T, Gershunov A, Benmarhnia T. Wildfire smoke impacts respiratory health more than fine particles from other sources: observational evidence from Southern California. Nat Commun 2021;12:1493. 10.1038/s41467-021-21708-0 33674571 PMC7935892

[2] California Air Resources Board. New analysis shows spikes of metal contaminants, including lead, in 2018 Camp fire wildfire smoke. Sacramento, CA: California Air Resources Board; 2021. https://ww2.arb.ca.gov/news/new-analysis-shows-spikes-metal-contaminants-including-lead-2018-camp-fire-wildfire-smoke

[3] Reid CE, Finlay J, Hannigan M, Physical health symptoms and perceptions of air quality among residents of smoke-damaged homes from a wildland urban interface fire. ACS EST Air 2025;2:13–23. 10.1021/acsestair.4c00258 39817255 PMC11730870

[4] Li T, Chen H, Fung JCH, Large presence of bromine and toxic metals in ambient fine particles from urban fires. Atmos Environ 2023;295:119554. 10.1016/j.atmosenv.2022.119554

[5] Environmental Protection Agency. Integrated science assessment (ISA) for lead (final report). Washington, DC: Environmental Protection Agency; 2024. https://assessments.epa.gov/isa/document/&deid=359536

